# A novel role for ATR/Rad3 in G1 phase

**DOI:** 10.1038/s41598-018-25238-6

**Published:** 2018-05-02

**Authors:** Cathrine A. Bøe, Tine W. Håland, Erik Boye, Randi G. Syljuåsen, Beáta Grallert

**Affiliations:** Department of Radiation Biology, Institute for Cancer Research, Oslo University Hospital, Oslo, Norway

## Abstract

Checkpoint kinases are important in cellular surveillance pathways that help cells to cope with DNA damage and protect their genomes. In cycling cells, DNA replication is one of the most sensitive processes and therefore all organisms carefully regulate replication initiation and progression. The checkpoint kinase ATR plays important roles both in response to DNA damage and replication stress, and ATR inhibitors are currently in clinical trials for cancer treatment. Therefore, it is important to understand the roles of ATR in detail. Here we show that the fission yeast homologue Rad3 and the human ATR regulate events also in G1 phase in an unperturbed cell cycle. *Rad3*Δ mutants or human cells exposed to ATR inhibitor in G1 enter S phase prematurely, which results in increased DNA damage. Furthermore, ATR inhibition in a single G1 reduces clonogenic survival, demonstrating that long-term effects of ATR inhibition during G1 are deleterious for the cell. Interestingly, ATR inhibition through G1 and S phase reduces survival in an additive manner, strongly arguing that different functions of ATR are targeted in the different cell-cycle phases. We propose that potential effects of ATR inhibitors in G1 should be considered when designing future treatment protocols with such inhibitors.

## Introduction

Genomic instability can drive cancer development and is a characteristic of most cancers. Cells have evolved numerous mechanisms to protect their genome. These include checkpoints, mechanisms that safeguard the genome by restricting cell-cycle progression in the face of DNA damage. One of the key proteins in checkpoint responses is ATR (ATM and Rad3-related), which belongs to the PIKK (phosphatidyl inositol 3’kinase-related kinases) protein family. ATR is activated by a number of DNA-damaging agents as well as stalled replication forks. The common theme leading to ATR activation is the generation of single-stranded DNA (ssDNA) during DNA repair or replication^1,2^. ATR is a serine-/threonine kinase that transduces the ssDNA-induced signal by phosphorylating several substrates, including the CHK1 kinase. In cells with damaged DNA, propagation of the signal results in induction of cell-cycle arrest, stimulation of DNA repair and stabilisation of stalled replication forks^3^.

ATR is essential in most organisms, suggesting a role also in an unperturbed cell cycle^4–6^. The essential role of ATR is not well understood, but budding yeast cells containing a mutant form of the ATR homologue *MEC1* can be rescued by deleting a suppressor of ribonucleotide reductase^7,8^, suggesting that the essential role of MEC1 is to regulate dNTP levels. Later, it was shown that ATR in higher eukaryotes controls the sequential activation of early and late replication origins during an unperturbed S phase^9,10^, regulates CDK activity through S phase^11^, limits the recruitment of And-1 - DNA polymerase alpha to GINS^12^ and is required for stabilizing stalled replication forks^13^. The general consensus regarding the role of ATR in unperturbed cells is that ATR activity is required in every S phase in response to the replication stress arising, which can be the source of endogenous DNA damage and may lead to constitutive low-level ATR activation. Regulation of origin firing through S phase or controlling dNTP levels are possible additional essential functions in higher eukaryotes^14^.

All these reports link ATR to important roles during S phase. However, preparation for DNA replication starts already in G1 phase as soon as cells exit mitosis, and involves induction of a transcriptional programme inducing expression of many of the genes encoding S-phase proteins, as well as assembly of replication complexes. This assembly of the replication complexes is performed in two separate stages to ensure that each replication origin is fired once and only once. First, the Pre-replicative complexes (preRC) are loaded onto future origins in early G1 phase. This involves loading of an inactive form of the core of the DNA helicase (MCM complex) onto chromatin in a CDC6 (Cdc18^Sp^)- and CDT1-dependent manner. Second, the CDK activity rises at the G1/S transition and the accessory components of the replicative helicase (CDC45 and GINS) are loaded onto the MCM core, forming the pre-initiation complex (preIC). Then the DNA is unwound allowing PCNA (proliferative cell nuclear antigen) to clamp onto DNA at primer-template junctions. The DNA polymerase can bind to PCNA and replication, and S phase, starts^15^. Even slight deregulation of any of the steps above leads to more replication stress during S phase, threatening genomic stability^16,17^. In cancer cells replication stress is increased, often due to increased CDK activity, which in turn influences the steps described above^18^. Increased replication stress enhances the dependency of cancer cells on ATR and CHK1. This dependency is further emphasized by the fact that ATR and CHK1 levels often are upregulated in neoplasms and are thought to promote tumour growth^19^. ATR is therefore seen as a promising target for cancer therapy and clinical trials exploiting specific ATR inhibitors (ATRi-s) for their cytotoxic effect are ongoing^20^.

We recently identified Hpz1 in fission yeast as a potential functional partner of Rad3, which is the fission yeast homologue of ATR^21^. Interestingly we found no evidence for Hpz1 participating in the checkpoint functions of Rad3. In the same study, we found that Hpz1 regulates cell-cycle progression from G1 to S phase; both preRC formation and bulk DNA replication started earlier in an *hpz1*Δ strain than in a wild-type strain when released from early G1 arrest. These findings pointed to Rad3, and probably its homologues, playing a role in the regulation of the G1/S transition.

Here, we report that both Rad3 in fission yeast and ATR in human cells have important function(s) in G1 and affect the initiation of DNA replication in an unperturbed cell cycle. The consequences of lost ATR activity in G1 phase are investigated. Our findings have implications for the use of ATRi-s in cancer therapy.

## Results

### Rad3 regulates cell-cycle progression through G1 phase in fission yeast

**Figure 1:**
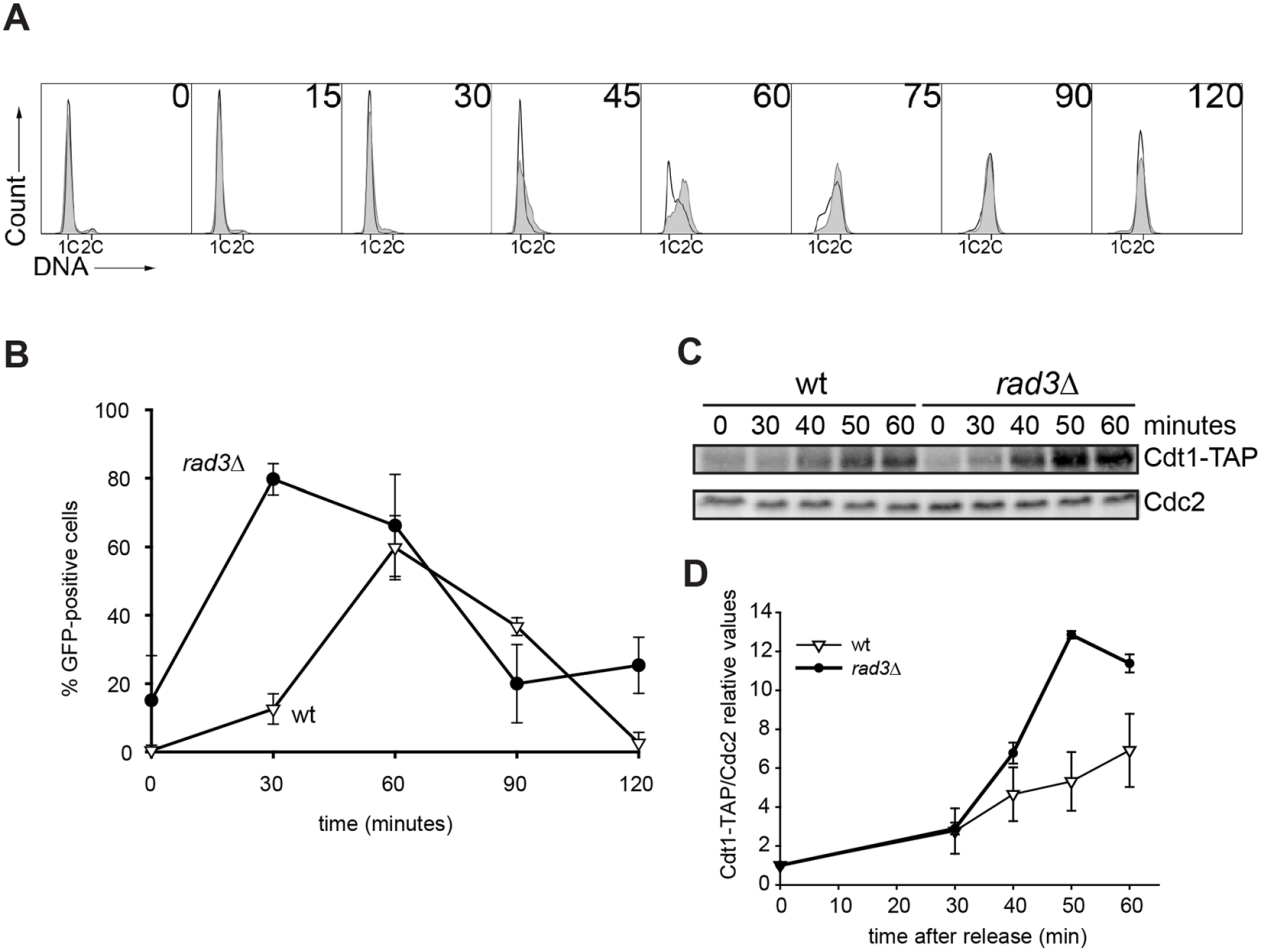
The importance of Rad3 for cell-cycle progression. Analysis of DNA content, preRC formation and Cdt1 levels in wild-type and *rad3*Δ cells synchronized in G1-phase, released into the cell cycle and incubated for the times indicated. (**A**) DNA histograms of individual wild-type (no fill) and *rad3*Δ (grey fill) cells. (**B**) Percentage of wild-type (∇) and *rad3*Δ (•) cells that contained chromatin-bound preRC (Mcm2-GFP). (**C**) Immunoblot showing the expression of Cdt1-TAP in total cell extracts. The presence of Cdc2 is shown as loading control. Full-length blots are presented in Supplementary Fig. S1. (**D**) Quantification of Cdt-TAP signal normalized to Cdc2 loading control. Average of three blots and SE are shown.

To investigate whether the fission yeast ATR homologue Rad3 has a role in G1 phase, we monitored the entry of *rad3*Δ cells into S phase after release from an arrest in early G1 phase (Fig. 1A). Already 45 minutes after release the *rad3*Δ cells have synthesized more DNA than wild-type cells and thus progressed further into the cell cycle. The time difference between *rad3*Δ and wild-type cells is about 15 minutes, which is a substantial difference, considering that the duration of G1 phase is about 15 minutes. To assess whether the difference observed is due to an effect in G1 or in early S phase, the loading of MCM2-GFP, a part of the MCM complex, onto chromatin was investigated (Fig. 1B). We observed that the loading of MCM2 was advanced in *rad3*Δ cells (Fig. 1B). One of the proteins required for loading the MCM complex onto chromatin is Cdt1^22^, whose expression is cell-cycle regulated and increasing through M and G1 phase until its degradation in S phase^22,23^. We analysed the Cdt1 level by immunoblot analysis in cells progressing through G1 phase (Fig. 1C). Remarkably, in the absence of Rad3 detectable levels of Cdt1 are present earlier, and reach peak expression earlier. Between 50 and 60 minutes after release into the cell cycle the amount of Cdt1 in the *rad3*Δ mutant decreased (Fig. 1D), probably because the cells by then have entered S phase (Fig. 1A) where Cdt1 is degraded. Thus, the kinetics of Cdt1 expression is consistent with the interpretation that *rad3*Δ cells are advanced in G1 phase of the cell cycle.

Taken together these results suggest that Rad3 negatively regulates progression through the G1 phase in *S. pombe¸* at a step at or prior to Cdt1 expression and preRC formation.

### The G1 role of Rad3 is conserved

**Figure 2:**
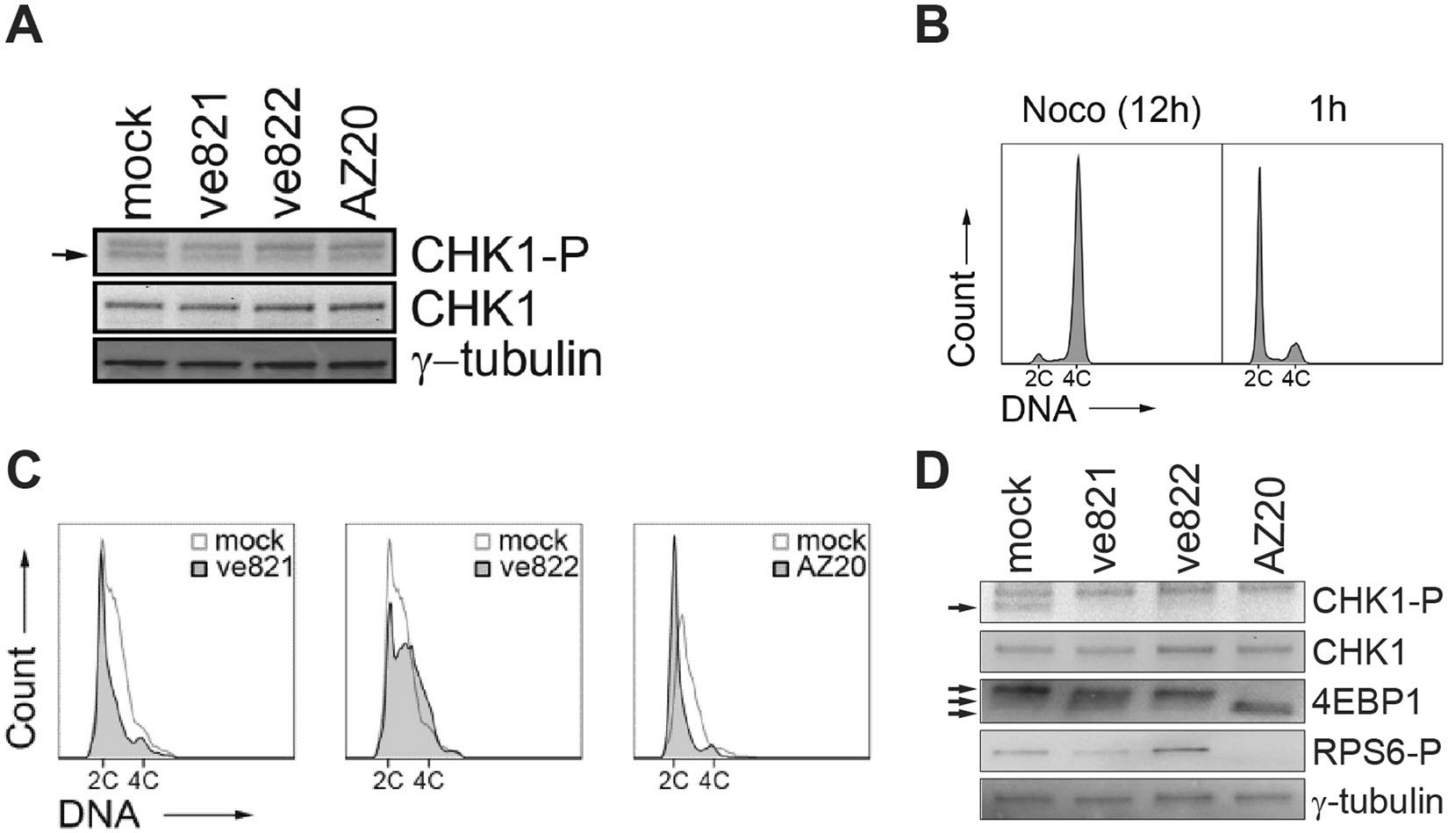
Three ATR inhibitors differently affect cell-cycle progression. (**A**) Immunoblot showing the presence of CHK1-P (S345) in total cell extracts of U2OS cells treated with ATRi’s ve821(10 µM), ve822 (160 nM) or AZ20 (3 µM) for 1 hour. Total CHK1 levels are shown for comparison and γ-tubulin is shown as loading control. Full-length blots are presented in Supplementary Fig. S2. (**B**) DNA histograms of U2OS cells synchronized in mitosis (nocodazole and shake off, left), released into the cell cycle and incubated for 1 h (right). (**C**) DNA histograms of U2OS cells synchronized in mitosis, released into the cell cycle and incubated with different ATRi’s (10 μM ve821, 160 nM ve822, 3 μM AZ20) and mock (DMSO) from 1 hour after release, samples were taken 11 h after release. DNA staining and flow cytometry analysis of the ATRi-treated samples were performed using barcoding, each set contained a mock-treated sample for normalization. (**D**) Immunoblot showing the presence of CHK1-P (S345), RPS6-P and 4EBP1 in total cell extracts of U2OS cells treated with ATR inhibitors ve821 (10 μM), ve822 (160 nM) or AZ20 (3 μM). The inhibitors were added 1 h after release from nocodazole arrest and shake off, for a duration of 4 h. Arrows point to several 4EBP1 bands. Total CHK1 levels are shown for comparison and γ-tubulin is shown as loading control. Full-length blots are presented in Supplementary Fig. S3.

The checkpoint functions of Rad3, ATR and their homologues are highly conserved. We investigated whether the phenotype of early entry into S phase in the absence of Rad3 was conserved from fission yeast to human cells. Since ATR is essential, we used ATR inhibitors to reduce ATR activity. We tested three different inhibitors and followed the level of CHK1 phosphorylation at position S345 in U2OS cells to assess ATR activity. The level of CHK1-P was reduced one hour after addition of each of the inhibitors (Fig. 2A), verifying efficient inhibition of the kinase activity of ATR. To determine the effect on cell-cycle progression of loss of ATR activity in G1 phase, U2OS cells were arrested in prometaphase by nocodazole treatment for 12 hours, collected by mitotic shake-off, and seeded into fresh medium. One hour after release into the cell cycle most cells had progressed into G1 (Fig. 2B) and the ATR inhibitors ve821 (10 µM), ve822 (160 nM) or AZ20 (3 µM) were added. Eleven hours later mock-treated cells (solvent only) had progressed into S phase as judged by their DNA content (Fig. 2C). We found that cells treated with either ve821 or AZ20 delayed bulk DNA replication, whereas ve822-treated cells had replicated more DNA than mock-treated cells (Fig. 2C). The opposite effects of the different inhibitors suggest off-target effects and we considered mTOR, another member of the PIKK family, as a possible target of ve821 and AZ20. Inhibition of mTOR delays the progression from G1 to S in mammalian cells^24,25^, just like treatment with ve821 or AZ20 does (above). To assess mTOR activity we monitored phosphorylation of 4EBP1 (direct substrate) and RPS6 (indirect target) in cells released from nocodazole and treated with the three ATR inhibitors for 4 hours in G1 phase. mTOR was clearly inhibited by ve821 or AZ20 as seen by reduced phosphorylation of both 4EBP1 and RPS6 (Fig. 2D). In contrast, mTOR was not inhibited in cells treated with ve822 (Fig. 2D). We conclude that at the concentrations required to obtain satisfactory ATR inhibition, ve821 and AZ20 inhibit mTOR, which masks the possible effect of ATR inhibition on G1 to S phase progression. However, ve822 does not inhibit mTOR and the advanced cell-cycle progression observed is most likely due to an effect of ATR inhibition in G1, consistent with the results obtained using the fission yeast *rad3*Δ mutant. In all the following experiments described here we used ve822 to inhibit ATR.

### Inhibition of ATR in G1 delays entry into S phase

As shown in Fig. 2C, inhibition of ATR reduces the amount of DNA that is replicated at a given timepoint. This could be due to an effect in G1 on preparation for DNA replication, such as formation of the preRC or the preIC. Alternatively, ATR has a well-established role in the regulation of origin firing^9,10,12^, and deregulated and earlier origin firing might appear as earlier entry in to S phase. To distinguish between these possibilities we monitored entry into S phase in ATRi-treated cells using four different approaches.

**Figure 3:**
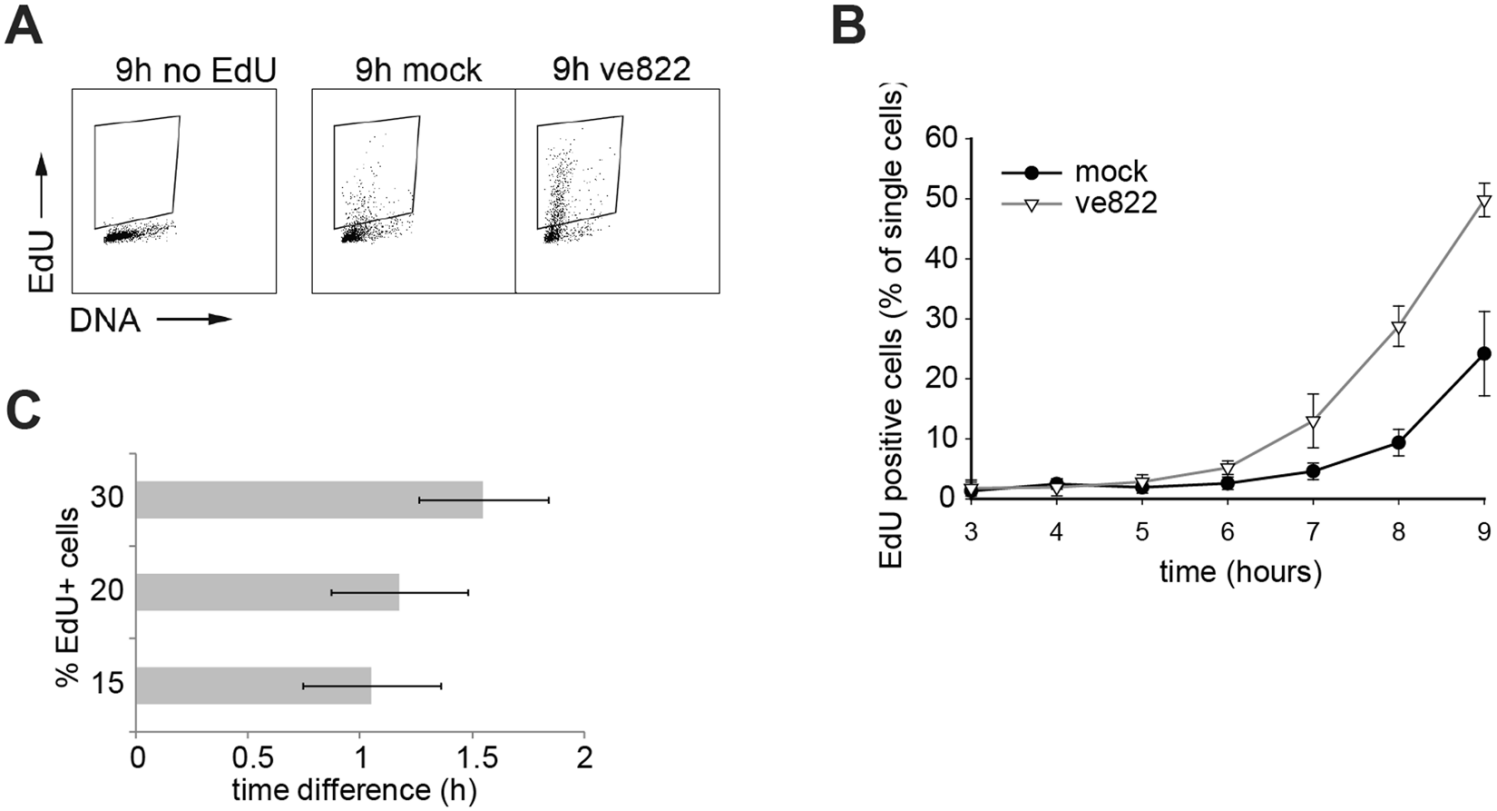
Cell-cycle progression in U2OS cells lacking ATR activity in G1 phase. (**A**) Dot plots showing DNA content versus EdU intensity in individual U2OS cells 9 hours after release from nocodazole-induced arrest. Cells were treated with 160 nM ve822 (∇) or mock (DMSO) (•) from 1 h onwards. (**B**) Quantification of EdU-positive U2OS cells at the indicated time points after nocodazole arrest and shake off. Cells were treated as in A. Error bars show standard deviations of three independent experiments. (**C**) Quantification of EdU-positive cells at the indicated time points after shake off of mitotic cells from exponentially growing U2OS cells (without nocodazole). 160 nM ve822 (∇) or mock (DMSO) were added 1 h after release, for 4 h. The experiment was repeated 4 times and the number of EdU-positive cells as a function of time was plotted in each experiment (a representative experiment is shown in Supplementary Fig. S4). Then the time-difference between the two curves at three different values of % EdU-positive cells was plotted. The average of four experiments and standard deviations are shown.

In the first approach, EdU was added to label the DNA at active replication forks. In cells synchronized by nocodazole and mitotic shake-off, EdU-positive cells started to appear at least one hour earlier after ve822 treatment than in untreated control cells (Fig. 3A,B). To confirm that this was not a consequence of the nocodazole treatment, exponentially growing cells were also synchronized by mitotic shake-off in the absence of nocodazole (Fig. 3C). The ATRi and EdU were added 1 h after shake-off and the number of EdU-positive cells was counted. Similarly to the results obtained after nocodazole-induced arrest, the EdU-positive cells appeared at an earlier time in the ATRi-treated cells (Fig. 3C). Since the start of EdU-incorporation can be interpreted as the start of S phase, these data argue that the difference is due to different rates of progression through G1 phase.

**Figure 4:**
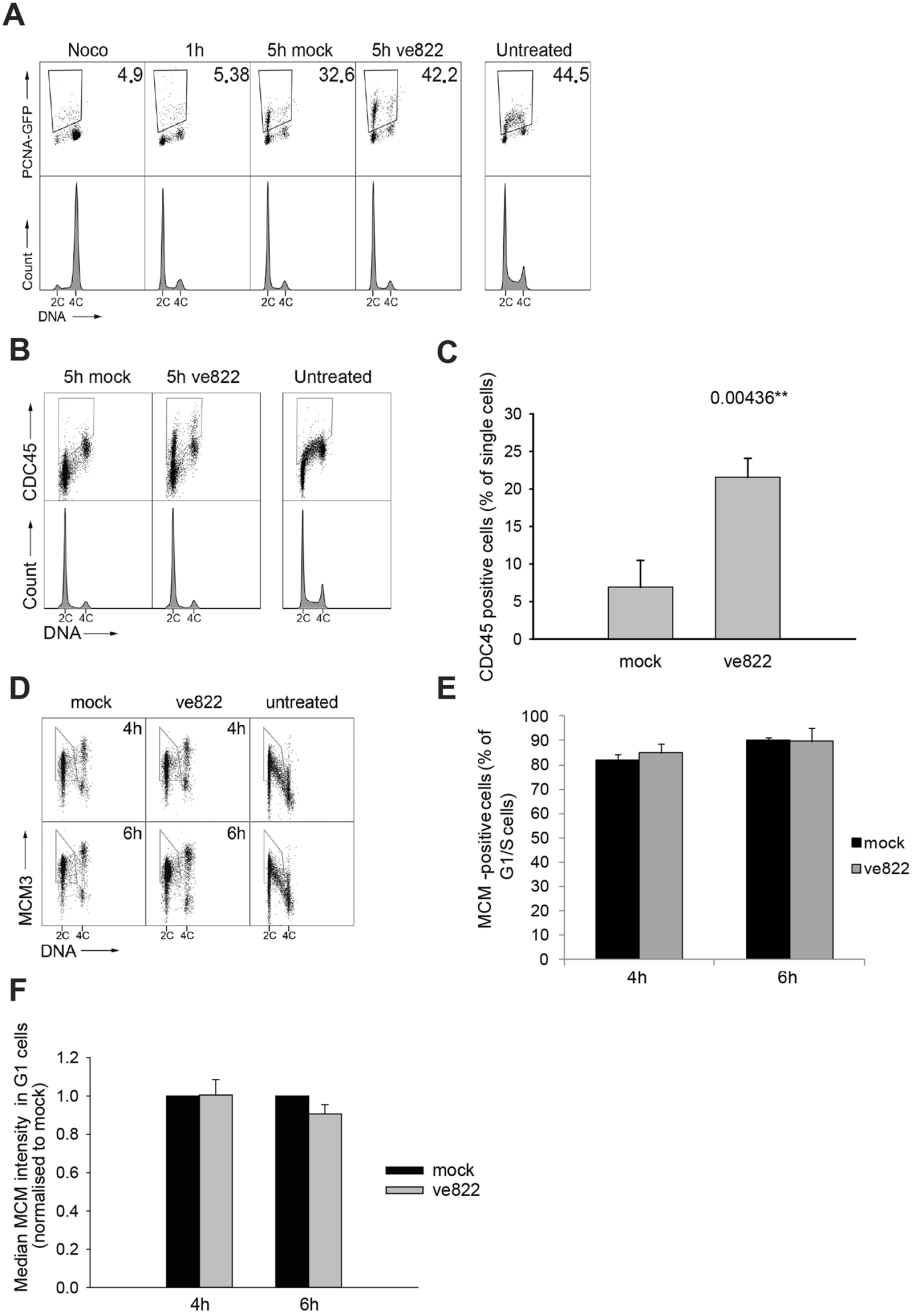
Chromatin loading of replication initiation factors after ATR inhibition in G1 phase (**A**) Dot plots showing DNA content versus intensity of chromatin-bound PCNA-GFP in individual U2OS cells at the indicated time points after release from nocodazole-induced mitotic arrest. Cells arrested and 1 h after release were used as negative controls to determine the region of interest. ATRi ve822 (160 nM) or mock (DMSO) was added 1 hour after release from the mitotic arrest. The staining for flow cytometry of arrested, 1 hour, 5 hour mock and 5 hour ve822 samples was performed in the same barcoding set. Numbers show the percentage of PCNA-GFP positive cells. The pattern of chromatin-bound PCNA-GFP in exponentially growing cells is shown for comparison. DNA histograms of each time point are shown for reference. (**B**) Dot plots showing DNA content versus intensity of chromatin-bound CDC45 in individual U2OS cells at the indicated time points after release from nocodazole-induced mitotic arrest. ATRi ve822 (160 nM) or DMSO (mock) was added 1 hour after release from the mitotic arrest. The samples shown were stained with antibodies in the same barcoding set. Supplementary Fig. S5 B shows how the CDC45-positive cells were identified. (**C**) Quantification of CDC45-positive U2OS cells shown in E. Error bars show standard deviations calculated from 3 independent experiments. The number represents the p-value from a two-tailed t-test. (**D**) Dot plots showing DNA content versus intensity of chromatin-bound MCM3 in individual U2OS cells at the indicated timepoints after release from nocodazole-induced mitotic arrest. ATRi ve822 (160 nM) or mock (DMSO) was added 1 hour after release from the mitotic arrest. The samples shown were stained with antibodies in the same barcoding set. The region of interest was determined from the pattern of MCM-loading in exponentially growing cells (“Untreated”). (**E**) Quantification of MCM-positive U2OS cells in G. Error bars show standard deviations calculated from 3 independent experiments. (**F**) Quantification of the MCM signal intensity in G1 cells in G. Error bars shows standard deviations calculated from 3 independent experiments. Supplementary Fig. S5C,D shows gating and DNA histograms.

In the second approach, we used an *in situ* chromatin binding assay to measure the extent of loading of PCNA-GFP, which is a prerequisite for the DNA polymerase to bind to chromatin and start DNA replication. Five hours after release from the nocodazole arrest, before EdU-positive cells start to appear, the number of PCNA-GFP-positive cells increased by 30% more in cells treated with ATRi than in mock-treated control cells (Fig. 4A), suggesting that ATRi interferes with an event in G1, before the cells enter S phase.

In the third approach, we measured the loading of CDC45 onto chromatin after extraction of unbound protein (see Supplementary Fig. S5 A), an event necessary for PCNA loading. CDC45 is part of the CMG helicase and loads onto chromatin from late G1 through S phase, after formation of the preRC. We observed that 5 hours after release from mitosis there was more CDC45 loaded per cell (mean intensity) and more CDC45-positive cells in the culture treated with the ATRi than in the mock-treated culture (Fig. 4B,C).

Finally, we compared chromatin binding of MCM in untreated cells and cells treated with ATRi in G1. We previously found that in the absence of ATRi MCM loading in U2OS cells gradually increased over the first 4 h in G1 phase after release from nocodazole-induced arrest^26^. Interestingly, here we observed no difference in either the number of MCM-positive G1/S cells or the median MCM signal intensity in G1 upon addition of ATRi (Fig. 4D,E and F) suggesting that preRC formation is not affected by ATR inhibition.

The earlier loading of PCNA and CDC45 as well as the higher number of EdU-incorporating cells upon ATR inhibition in G1 strongly suggests that ATR regulates an early event upstream of CDC45 loading in the sequence of events leading up to origin firing. However, in contrast to in fission yeast, preRC loading is not affected in human cells. Thus, while the G1 function of Rad3 in fission yeast and ATR in mammalian cells seems to be conserved in that both affect a step upstream of replication origin firing, they most likely have different molecular mechanisms.

### Effect of ATRi on G1-S progression is independent of synchronization method or choice of cell line

**Figure 5:**
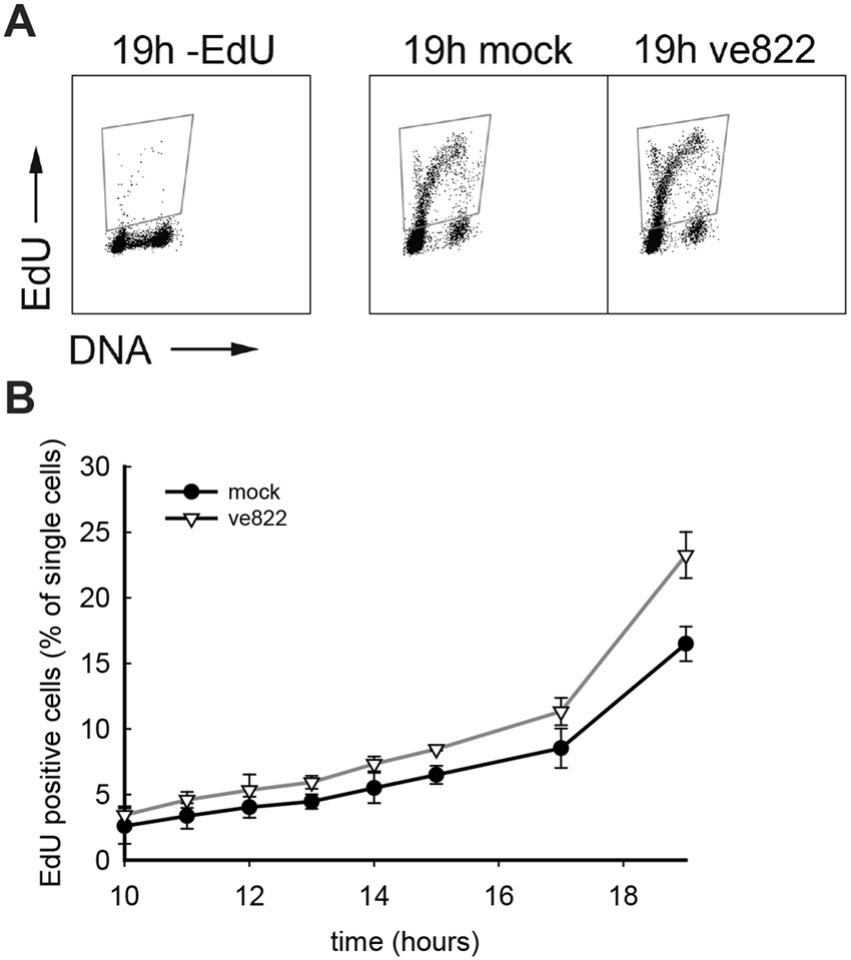
Importance of ATR in RPE cells in G1. Analysis of EdU incorporation in RPE cells synchronized in G0, released into the cell cycle and treated with ATR inhibitor in G1. (**A**) Dot plots showing DNA content versus EdU intensity in individual RPE cells 19 hours after release from G0. Gating for EdU-positive cells was determined based on the control without EdU. ATRi ve822 160 nM was added at the time of release from G0 arrest. (**B**) Quantification of EdU-positive RPE cells at the indicated time points of cells treated with 160 nM ve822 (∇) or mock (•) as in A. Each time point was performed in separate barcoding sets including a sample from exponentially growing cells not given EdU (-EdU control). Error bars show standard deviations calculated from 3 independent experiments.

To investigate whether the effect of ATR inhibition during G1 is present also in non-cancerous cells and after other synchronization methods, we examined the effect in hTERT RPE (human TERT retinal pigment epithelial) cells synchronized in G0 by contact inhibition and subsequent release (Fig. 5A,B). The synchrony obtained was not as good as after nocodazole arrest, as some cells had incorporated EdU already at the first time point, 10 hours after release. However, the vast majority of the cells appear to be progressing synchronously as judged from the sharp increase of EdU-positive cells. As the cells moved from G0 through G1 and into S phase the number of cells that have entered S phase (EdU-positive) was higher when cells were treated with the inhibitor than in the untreated control, although the difference was not as substantial as for U2OS cells (Fig. 3B). The slope of the curve is steeper when ATR is inhibited (Fig. 5B) and this indicates that cells are moving into S phase faster compared to mock-treated cells. We also analysed BJ cells (human foreskin fibroblasts) synchronized in G0 and obtained similar results as for hTERT RPE cells (Supplemenatary Fig. S6). Our data on these nontransformed cells strongly suggest that there is a role for ATR in G1, regulating initiation of DNA replication, even in the absence of any DNA-damaging or replication-stress-inducing drugs.

### ATR inhibition in a single G1 phase results in reduced survival

**Figure 6:**
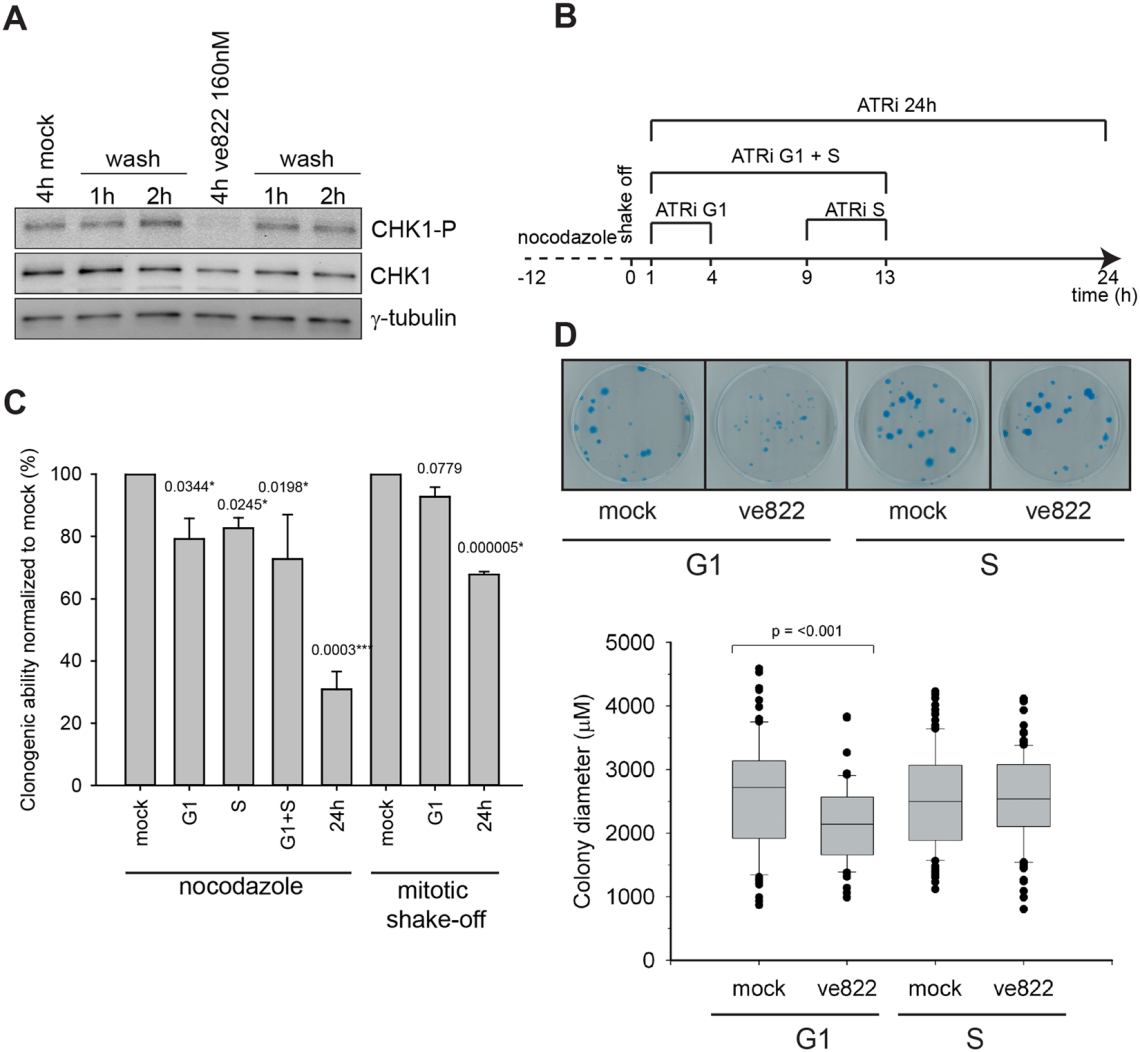
Responses to ATR inhibition in G1 phase. (**A**) Immunoblot showing the presence of CHK1-P (S345) in total cell extracts of U2OS cells treated with ATRi ve822 (160 nM) in G1. Inhibitor or DMSO (mock) was added 1 hour after release and washed out 4 hours later by exchanging the medium with fresh medium 3 times. Cells were harvested at the end of ATR inhibition (4 hours) and 1 and 2 hours after removal of inhibitor (wash). Total CHK1 levels are shown for comparison and γ-tubulin is shown as loading control. Full-length blots are presented in Supplementary Fig. S7. (**B**) Schematic description of the experiment shown in C and D. (**C**) Clonogenic survival of U2OS cells after inhibition of ATRi in one G1- or S-phase, G1 and S-phase combined or 24 hours as illustrated in B. Treatment was applied 1 hour after seeding and release from mitotic arrest (G1, G1 + S and 24 hour treatment). S-phase treatment was applied 9 hours after release. After treatment inhibitor or DMSO (mock) was removed as denoted above in A and the dishes were cultured further for 14 days. Relative survival is normalized to 100% for mock. Error bars are indicated as standard deviations of three independent experiments. Numbers are p-values obtained from a two-tailed t-test. (**D**) Photographs show representative dishes from each treatment. Box plots show colony diameter of U2OS cells after the clonogenic assay performed in B. The colonies were measured 14 days after release from nocodazole arrest and treatment. Horizontal bars are median, upper and lower edges of box are at 75th and 25th percentiles, lines extending from box are 90th and 10th percentiles. Single dot shows 95^th^ and 5^th^ percentiles. Each box represents 70–110 measurements. Median latencies between groups mock (G1) and ve822 (G1) were 2715.28 and 2139.61 µM; the distributions in the two groups differed significantly (Mann–Whitney *U* = 2225, *n*_1_ = 68 *n*_2_ = 95, *P* < 0.001).

The earlier transition from G1 to S phase could lead to problems during DNA replication if, for instance, factors required for replication are in short supply. Stalling of the replication machinery due to such shortage can lead to DNA damage^27^. Therefore, we wished to examine the consequences for cell survival after ATR inhibition in G1 phase. First, we examined whether ATR inhibition in G1 is reversible (Fig. 6A). We synchronized U2OS cells by nocodazole arrest (12 hours) followed by mitotic shake-off. One hour later, as cells entered G1 (Fig. 2B), ATRi or DMSO (mock) was added. To avoid ATR inhibition in early S phase the inhibitor was removed 4 hours later, before the first S-phase cells were detected (as seen in Fig. 3B). The presence of CHK1-S345 phosphorylation was analysed after 4 hours of treatment with ATRi and then one and two hours after washing out the inhibitor. ATR-dependent CHK1-S345 phosphorylation is clearly inhibited after 4 h of treatment with ATRi and this inhibition is reversible since CHK1-S345 phosphorylation is again observed, and similarly to that in mock-treated cells, one hour after removal of inhibitor (Fig. 6A). This finding allows us to study the effects of ATR inhibition through one G1 phase in synchronized cells. We used a clonogenic assay to investigate how the loss of ATR activity in a single G1 phase affects survival. U2OS cells synchronized by nocodazole were released into the cell cycle and subjected to ATR inhibition in either G1 or S phase, G1 plus S phase combined, or maintained for 24 hours before removal (Fig. 6B). Inhibition through G1 or S phase both led to a marked reduction in survival (~20%) compared to no inhibitor (Fig. 6C). In addition, we observed that combined inhibition through G1 and S phase decreased survival more than in either phase alone, indicating that there is an additive effect of ATR inhibition in G1 and S phase. For comparison, we also investigated the effect of ATR inhibition in the first G1 phase of cells released from mitotic shake-off (no nocodazole). Reduced survival after treatment with ATRi was also observed in these cells, although not as pronounced as after nocodazole arrest. The impact on survival was less severe both when ATR was inhibited in G1 only or for 24 h.

To our surprise we noted that the colonies were smaller after ATR inhibition in G1 phase than without inhibition or with ATR inhibition in S phase (Fig. 5D). The difference in colony diameter was statistically significant as determined by a Mann Whitney U-test (p =  < 0.001). The reduction in colony size indicates that ATR inhibition in G1 phase has a cytostatic effect in addition to the cytotoxic effect.

**Figure 7:**
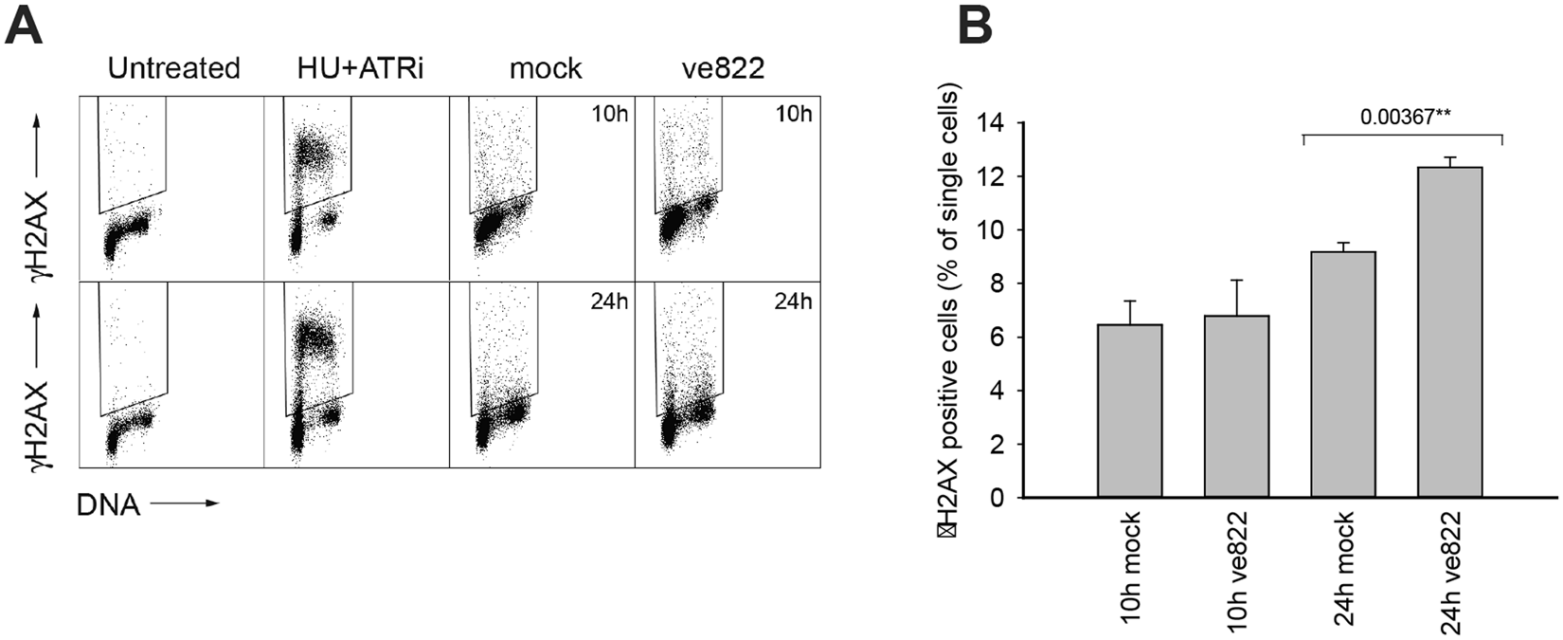
DNA damage upon ATR inhibition in G1 phase (**A**) Dot plot showing DNA content versus γH2AX intensity in individual U2OS cells 10 or 24 hours after release from mitotic arrest. ATRi ve822 160 nM was added 1 hour after release from the mitotic arrest and removed 4 hours later (see 5 A). Each time point was stained in a barcoding set that also included a positive (2 hours 2 mM HU + 160 nM ve822 in exponentially growing cells) and negative control sample to allow correct gating of γH2AX-positive cells. (**B**) Quantification of γH2AX-positive cells shown in D. Error bars show standard deviations of three independent experiments. Numbers represent p-values from a two-tailed t-test.

Having established that inhibition of ATR in G1 is deleterious for the cells, we investigated whether it induces DNA damage and/or apoptosis. To assess DNA damage, the amount of γH2AX (phosphorylated H2AX-S139) was assayed in cells treated with ATRi in G1 and harvested in the subsequent S phase or the next day (Fig. 7A,B). The fraction of γH2AX-positive cells was similar in ATRi- and mock-treated cells at 10 hours (S phase). However, 24 hours after treatment in G1 phase, the γH2AX-positive fraction was significantly increased (Fig. 7), suggesting increased DNA damage. For comparison, we exposed cells to the replication inhibitor hydroxyurea (HU) in the presence of the ATRi to induce severe DNA damage and assayed the amount of γH2AX. In the cells treated with the ATRi in G1 the level of γH2AX staining was much lower (Fig. 7A), which is not likely to induce apoptosis^28^. Consistently, we did not detect any increase in the number of apoptotic cells using the TUNEL assay 24 and 48 hours after ATR-inhibition in G1 (data not shown).

## Discussion

ATR (Rad3^Sp^) is known as a key protein in safeguarding the genome. It plays important roles in the response to DNA damage and it is also required in S phase in unperturbed cells. Impaired DNA-damage responses and increased replication stress are often associated with tumourigenesis, and, at the same time, the higher level of DNA damage also makes cancer cells more reliant on ATR-dependent stress responses than normal cells are. Consequently, cancer cells are particularly vulnerable to ATR inhibitors and they are being developed as cancer therapeutics. Therefore, a detailed understanding of the roles of ATR is required. Here we report an important novel function for ATR in the G1 phase of the cell cycle. We show that Rad^Sp^ and ATR regulate G1 events regulating the timing of S-phase entry and this function is important for cell survival. These results have important implications for the use of ATR inhibitors in therapy.

The early-replication phenotype of the fission-yeast *rad3*Δ mutant (Fig. 1) strongly suggests that Rad3 holds a function during the G1 phase of the cell cycle, affecting the initiation of DNA replication. We have shown that Rad3 affects steps at or prior to Cdt1 expression and MCM loading. Cdt1 expression in G1 is induced by the MBF (MluI-binding factor) transcription factor complex and the protein is degraded in S phase^23,29,30^. Earlier expression of Cdt1 in the *rad3Δ* mutant suggests that Rad3 delays the initiation of DNA replication by regulating MBF in G1. There are several reports in the literature showing regulation of the MBF complex by checkpoint proteins after stalled replication or DNA damage^31–33^. Our results suggest that Rad3 can regulate MBF-mediated transcription in G1, at least when restarting after a *cdc10-*induced arrest and possibly also in cycling cells. This function, in turn, affects timing of the G1-events required for DNA replication to commence in S phase. Similarly to the phenotype of the fission yeast *rad3*Δ mutants, inhibition of ATR activity in human cells also results in premature entry into S phase. The fact that DNA replication started earlier in U2OS, hTert RPE and BJ cells upon ATR inhibition in G1 phase argues that the effect is general and not cell- line specific. However, unlike in fission yeast, ATR in mammalian cells appears to regulate steps after MCM loading, but prior to CDC45 loading, delaying CDC45 and PCNA loading at the G1/S border (Fig. 2).

Previous studies have shown that ATR can be activated in G1 in response to DNA damage^34,35^ and that it has a role in unperturb**e**d S phase^9,10,12,36,37^. However, a role for ATR in G1 in unperturbed cells has not been described. ATR is involved in the regulation of origin firing in S phase^9,10,12,38^, and increased origin firing might appear as earlier entry into S phase. The following pieces of evidence support the notion that the early-replication phenotype is indeed due to inhibiting ATR function in G1 and not in S phase: (i) In fission yeast, Rad3 affects origin licensing (preRC formation), which can take place only in G1 and not in S phase. (ii) In human cells, the early-replication phenotype and the reduced survival can be observed when the ATR inhibitor is present for only 4 hours after synchronization, ie only in G1 (Figs 3C and 6B,C). It is most unlikely that in these experiments the effect on DNA replication is due to continued lower ATR activity in S phase, since ATR inhibition appears to be highly reversible as judged from the rapid CHK1 phosphorylation upon washing out the inhibitor (Fig. 6A). (iii) The preparation for DNA replication is affected, involving Cdc45 and PCNA loading. (iv) ATR inhibition in G1 reduces survival to the same extent as ATR inhibition in S phase, demonstrating that long-term effects of ATRi during G1 are as deleterious for the cell as ATR inhibition during S phase. Furthermore, the additive effect of ATR inhibition through both G1 and S phase argues that different functions of ATR are targeted and that ATR has an important function in G1. While our results clearly show that the observed early entry into S phase is a consequence of ATR inhibition in G1 rather than in S, future studies are needed to explore whether the G1 function of ATR regulates timing of entry into S phase directly or indirectly.

ATR has previously been linked to the activation of the spindle assembly checkpoint^39^ and was recently reported to have a function in chromosome segregation during mitosis^40^. Therefore, we considered the possibility that the apparent G1 function reported here reflects a requirement for ATR during the nocodazole arrest. However, early entry into S phase after ATR inhibition can be observed also after mitotic shake-off (without nocodazole) (Fig. 3C). Furthermore, the ATR inhibitor was added one hour after release form the nocodazole arrest, when the cells have completed mitosis and cytokinesis (Fig. 2B). These results strongly support the interpretation that the early entry into S phase is indeed a consequence of ATR inhibition in G1.

Correct duplication of the genome requires precise regulation of many steps so that all the factors are present at the right time, place and in the right amount. Therefore aberrant regulation in G1, when the cells prepare for DNA replication, will most likely cause increased replication stress and possibly more DNA damage during S phase. Surprisingly, we detected only a modest increase in DNA damage after ATR inhibition in G1, which is difficult to reconcile with the pronounced decrease in survival, and indicates that the novel function of ATR in G1 goes beyond affecting DNA replication. ATR is thought to be essential for cycling mammalian cells because of its function in S phase, but our results demonstrate that the unknown G1 function is of similar importance.

In addition to resulting in poor survival, ATR inhibition in G1 phase had a cytostatic effect not observed when inhibiting ATR during S phase (Fig. 5C). The mechanism behind the cytostatic effect requires further investigation. This surprising finding is highly relevant for the use of ATR inhibitors in cancer therapy and the cytostatic effects should be taken into account when future clinical trials employing ATR inhibitors are performed.

A pertinent question is how ATR is activated during an unperturbed G1 phase. The classic mechanism for ATR activation is recruitment to sites of ssDNA covered by replication protein A. ssDNA is generated at each replication fork and as DNA-repair enzymes process DNA damage. It is not immediately obvious how ssDNA is generated during an unperturbed G1 phase, but it is tempting to speculate that it is produced during transcription. In mitosis, bulk transcription is low and many transcription factors, as well as RNA Pol II, are excluded from chromatin^41^. Cells entering G1 have to “restart” transcription to make the proteins necessary for S-phase entry and DNA replication. Indeed, a recent study showed a spike of transcription at the mitosis-G1 transition^42^. This sudden increase in transcription is likely to result in a certain level of transcriptional stress. ssDNA created during transcription could pose a risk for genomic integrity^43^. There are several studies that report a requirement for ATR-mediated signaling upon stalling of the RNA polymerase, also in the absence of DNA lesions^44–47^. ATR is proposed to possess a protective role during transcription and we speculate that this role could be of particular importance in early G1 when there is a sudden increase in transcription. Interestingly, in S phase ATR ensures that replication slows down when encountering lesions. An analogous role during transcriptional stress in G1 would result in a shortened G1 phase when ATR is inhibited; the very phenotype we report here. Whether ATR couples the transcriptional wave to replication initiation through regulation of transcription alone or also by direct regulation of replication initiation factors is an interesting subject for future studies.

In conclusion, we have shown in both *S. pombe* and mammalian cells that lacking Rad3/ATR activity in G1 phase enables less restrictive S-phase entry. Deregulated S-phase entry is often accompanied by increased replication stress and can result in genomic instability. Treating patients with an ATR inhibitor could therefore create harmful effects also in normal cells, but as long as other components of the DNA-damage response are intact, such damage would be minimal. Our results support the idea that ATR inhibition is an efficient anti-cancer strategy and demonstrate an important novel aspect of ATR function in G1. This unknown G1 function should be considered both when designing combination-treatment strategies and when investigating unwanted side effects of ATR inhibitors.

## Methods

### Yeast strains, cell handling, staining and extraction

The *S. pombe* strains used in this study (Table S1), were derivatives of *Schizosaccharomyces pombe* L972 h-. Media and conditions were as described^48^. Cells were grown exponentially in Edinburgh minimal medium to an OD_595_ of 0.1–0.2 (2–4 × 10^6^ cells/ml). Synchrony in G1 phase was obtained by incubating the temperature-sensitive *cdc10-M17* mutant^49^ at 36 °C for 4 hours before they were released from the arrest into the cell cycle at 25 °C. Sytox green was used to stain cells for flow cytometry as described previously^50^. Extraction of unbound MCM2-GFP was performed as described^51^.

### Cell culture and synchronization

Human U2OS osteosarcoma and BJ fibroblast cells were cultivated in DMEM (Dulbecco’s Modified Eagle’s Medium) (Invitrogen) supplemented with 10% fetal bovine serum (FBS) (Gibco) and 1% Penicillin/Streptomycin (P/S) (Gibco). Human RPE epithelial cells immortalized with hTERT were cultivated in DMEM/F12 Glutamax supplement (Invitrogen) supplemented with 10% FBS, 1% P/S and 0.01 mg/ml Hygromycin B (Sigma). U2OS cells stably expressing pIRES-eGFP-PCNA were generated by transfection using Fugene HD (Roche) and selection for 3 weeks with puromycin (1 µg/ml). Synchronization of U2OS cells in prometaphase was performed by addition of 0.04 µg/ml nocodazole (Sigma-Aldrich) for 12–13 h. Mitotic cells were collected by shake-off and washed three times with regular medium before release. BJ and RPE cells were synchronized in G0 by growing the cells to 100% confluence followed by addition of fresh culture medium and cultivation for an additional 3 days. To release the cells from G0 phase the cells were subcultured at low density. For synchronization by mitotic shake-off U2OS cells were collected by shake-off and released into fresh medium. To inhibit ATR activity the ATRi-s ve821 (Axon Medchem), ve822 (Selleckchem) or AZ20 (Selleckchem) were dissolved in DMSO and added to the cells at a 1:1000 dilution. To induce γH2AX-positive cells 2 mM HU (Sigma) + 160 nM ve822 was added to the cells for 2 hours. The Click-iT EdU Alexa Flour 488 Flow Cytometry Assay Kit (Invitrogen) was used for measurements of EdU incorporation. EdU was added at the time of release from G0 or prometaphase to a final concentration of 1 µM. The TdT kit (Roche), biotin-16-dUTP (Roche) and Streptavidin-Cy5 (GE Healthcare) were used for the TUNEL assay according to the manufacturer’s instructions.

### Extraction and fixation

For extraction of not-chromatin-bound proteins cells were trypsinized and treated with 750 µl low salt extraction buffer (0.1% Igepal CA-630, 10 mM NaCl, 5 mM MgCl_2_, 0.1 mM PMSF, 10 mM Phosphate buffer (pH 7.4)) for 5 min on ice. Fixation was performed by addition of 10% formalin (HT501128 Sigma) to a final concentration of 2.5% and incubation for 1 hour on ice. Then the cells were washed with 1 × cold PBS^26^. For measurements of γH2AX, EdU or DNA-content cells were permeabilised and fixed with 1 ml ice-cold 70% EtOH after trypsination.

### Flow cytometry

To eliminate variations in antibody staining between individual samples, barcoding with pacific blue was performed^26,52^. Following barcoding the samples were incubated with primary antibodies (α-phospho-H2AX S139 (1:500, 05–636, Millipore), α-CDC45 (1:100, sc-55569, Santa Cruz Biotechnology) or α-MCM3 (1:200, N-19 sc-9850, Santa Cruz Biotechnology)) and secondary antibodies (α-mouse IgG Cy3 (1:250, Sigma) or α-goat IgG Alexa-Flour 488 (1:500, Life Technologies)) diluted in flow buffer (0.1% Igepal CA-630, 6.5 mM Na_2_HPO_4_, 1.5 mM KH_2_PO_4_, 2.7 mM KCl, 137 mM NaCl, 0.5 mM EDTA (pH 7.5)) containing 5% nonfat milk. Fx cycle Far Red (Life Technologies) was used for DNA staining. Flow cytometry was performed on an LSRII flow cytometer (BD Biosciences).

### Immunoblots

Samples for immunoblots of mammalian proteins were prepared by 3 × wash with cold 1 × PBS and kept at −80 °C until 2 × Laemmli buffer was added to make whole cell lysates. Total cell extracts for immunoblots of *S. pombe* proteins were made by TCA protein extraction^53^. Antibodies for immunoblots were α-γtubulin (1:10000, T6557, Sigma-Aldrich), α-Chk1-P345 (1:1000, 2348, Cell Signaling Technology), α-CHK1 (1:200, DCS310.1, Santa Cruz), α−4EBP1 (1:2000, Cell Signaling Technology) α-phosphoAkt substrates (1:1000, 23C8D2, Cell Signaling Technologies), α-PSTAIRE, recognizing a motif in cdc2 (1:2000, Santa Cruz Biotechnology sc-53) and anti-peroxidase PAP1, against the TAP-tap (1:1000, Sigma P1291). Appropriate ECL and ECF kits were used for detection.

### Clonogenic survival assays

200 cells were seeded into each 6 cm dish. In each experiment 3 dishes were seeded for each treatment. The cells were cultured for 14 days, fixed in 70% EtOH and stained with methylene blue. Colonies containing more than 50 cells were counted as survivors. Survival fractions were calculated for each experiment (performed 3 times) and the average and standard deviations were calculated. To measure colony diameters experiments were performed in 6 well plates seeding 100 cells/well and a GelCount^TM^ from Oxford Optronix was employed.

## Electronic supplementary material

Supplementary info
