# Supplementary material for: A novel role for ATR/Rad3 in G1 phase
Source: Sci Rep. 2018 May 2;8:6880. doi: 10.1038/s41598-018-25238-6 (PMC5931961; doi:10.1038/s41598-018-25238-6)
Supplement: Supplementary file 1 — Supplementary info [file 41598_2018_25238_MOESM1_ESM.pdf]

# A novel role for ATR/Rad3 in G1 phase

Cathrine A Bøe, Tine W Håland, Erik Boye, Randi G Syljuåsen, Beáta Grallert*

Department of Radiation Biology, Institute for Cancer Research, Oslo University Hospital, Oslo, Norway

**Corresponding author**: Beáta Grallert

Email beata.grallert@rr-research.no

Tel +4722781980

**Supplementary Figure S1**

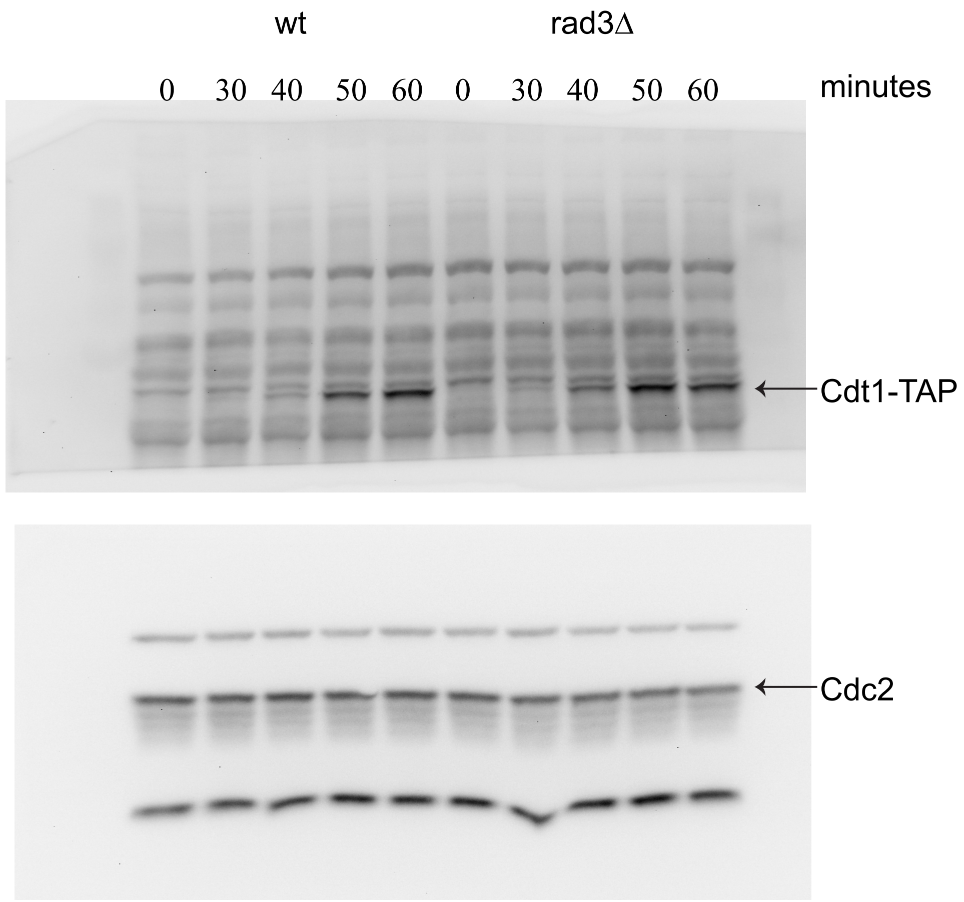

**Supplementary Figure S2**

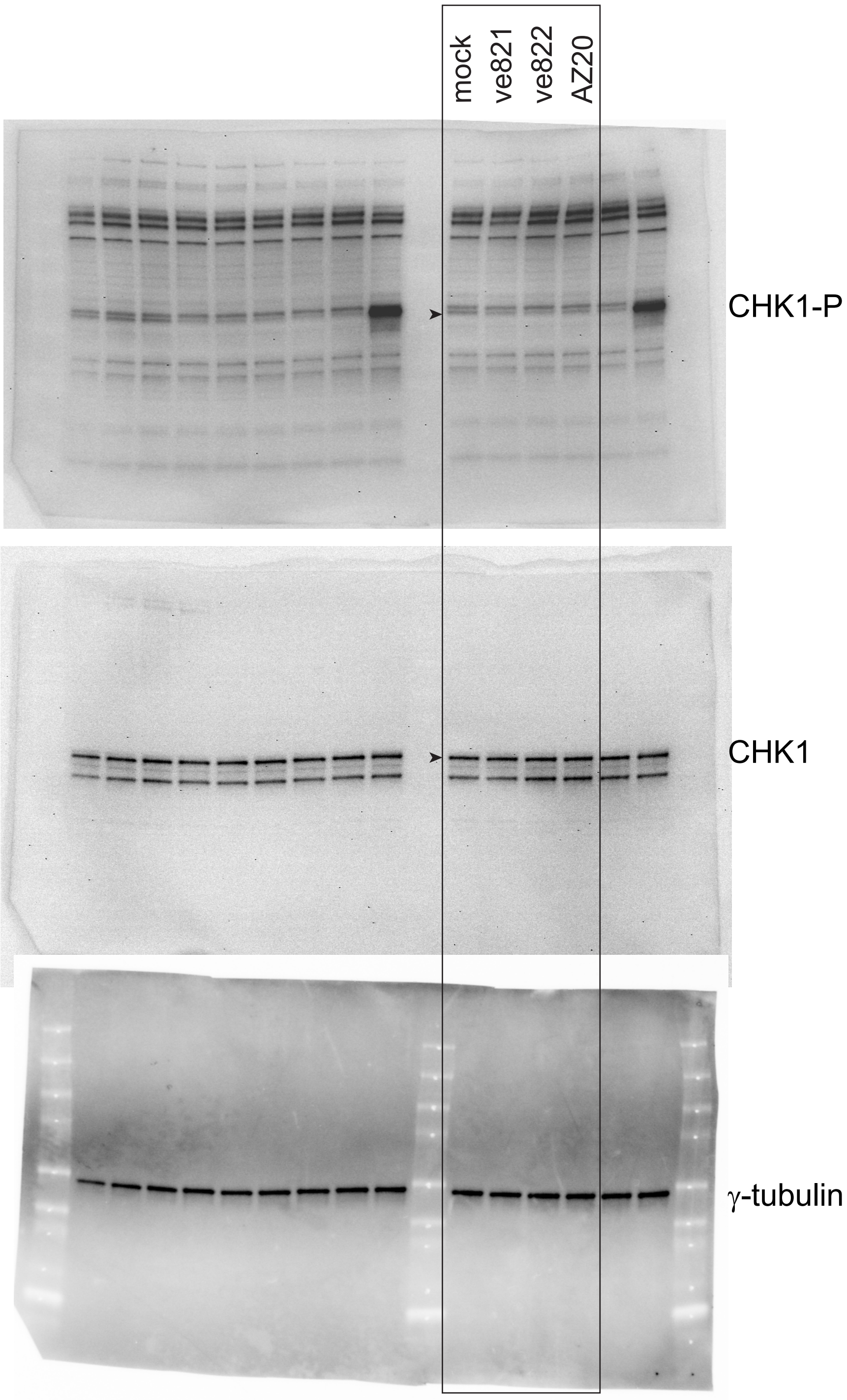

**Supplementary Figure S3**

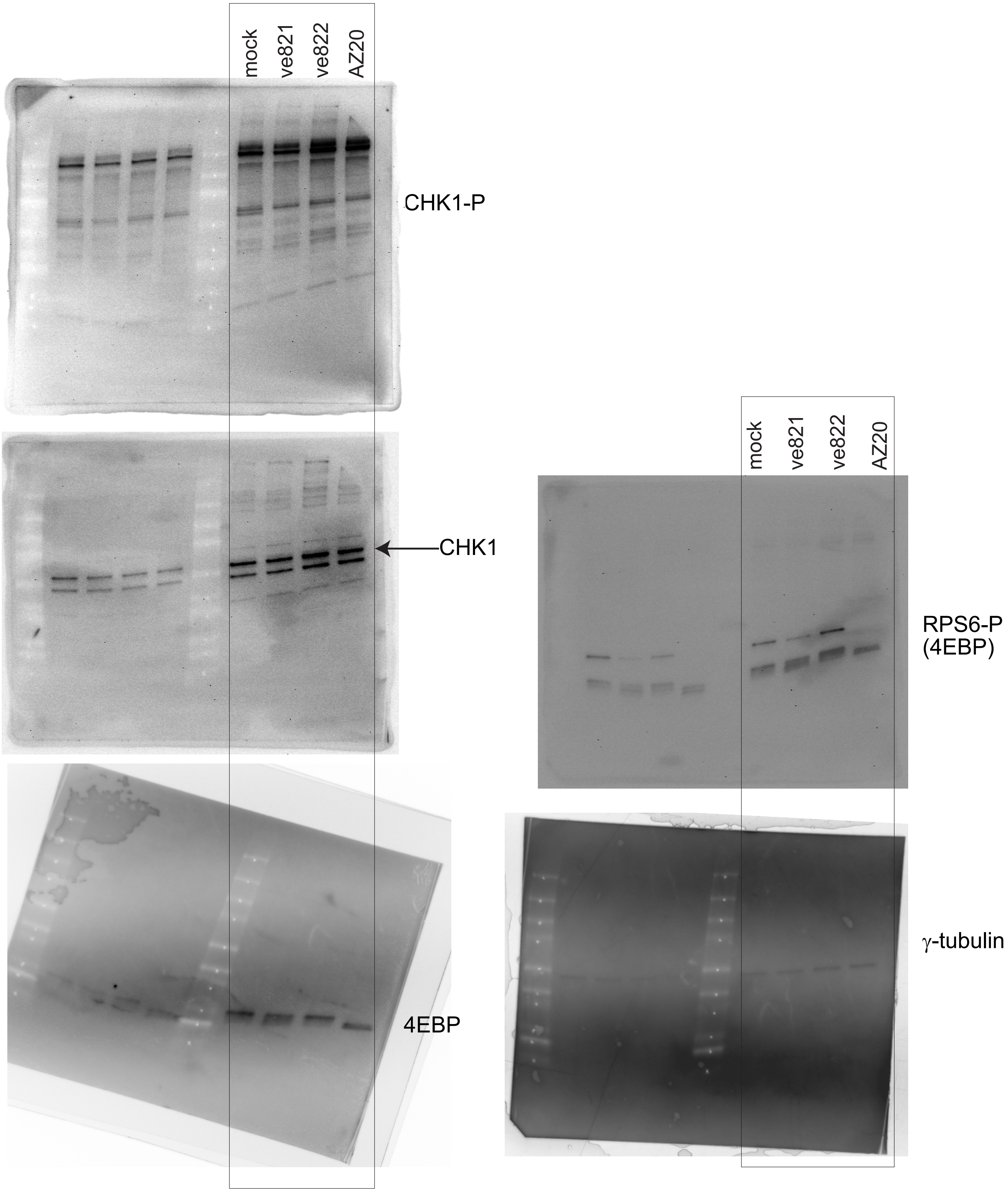

## Supplementary Figure S4

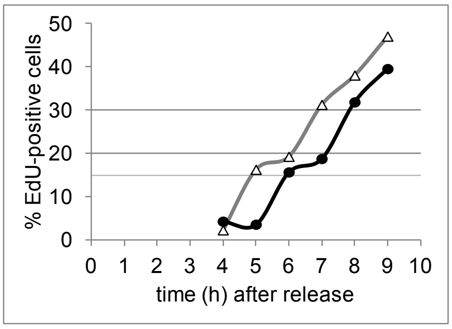

**Supplementary Figure S5**

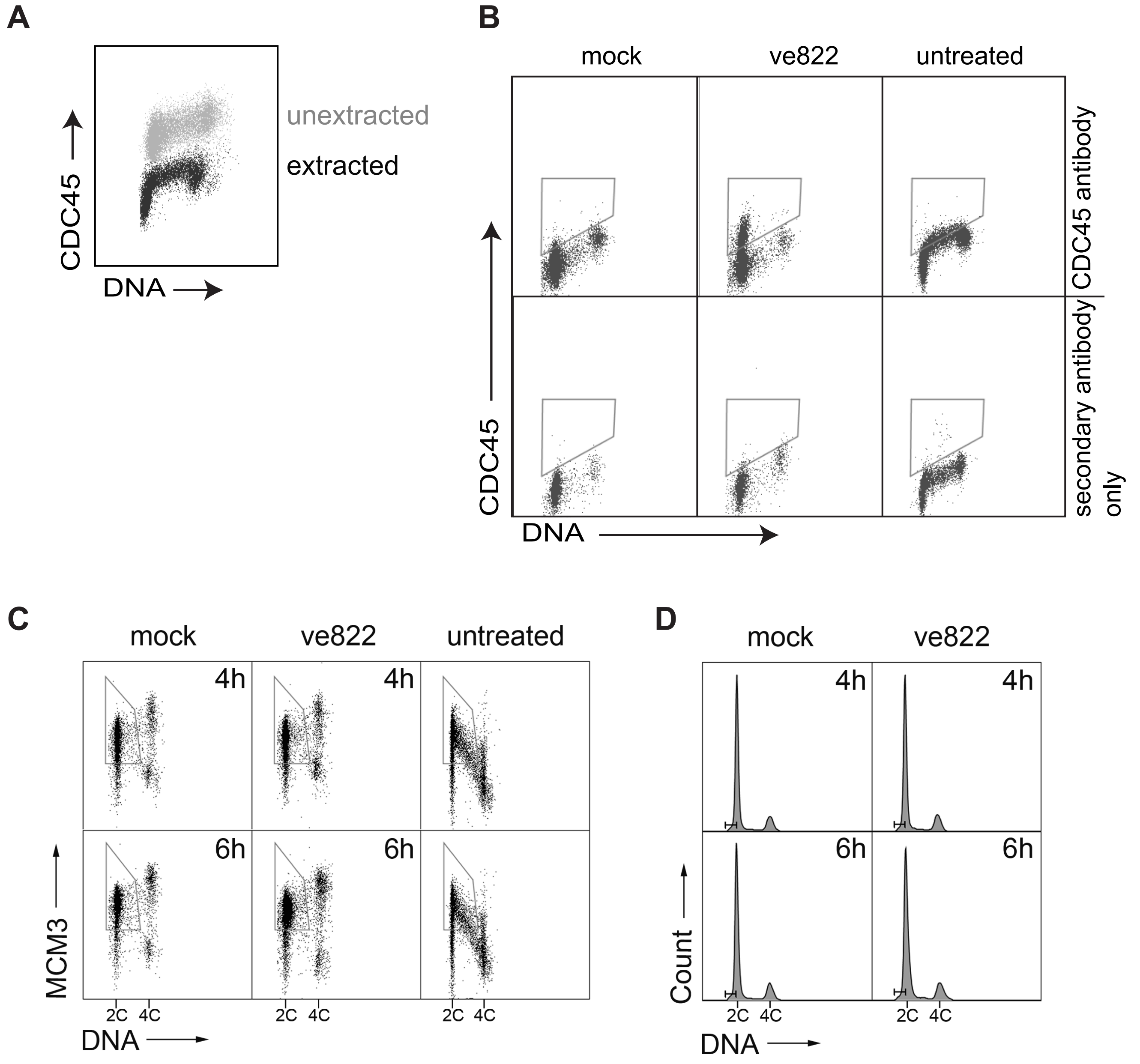

**Supplementary Figure S6**

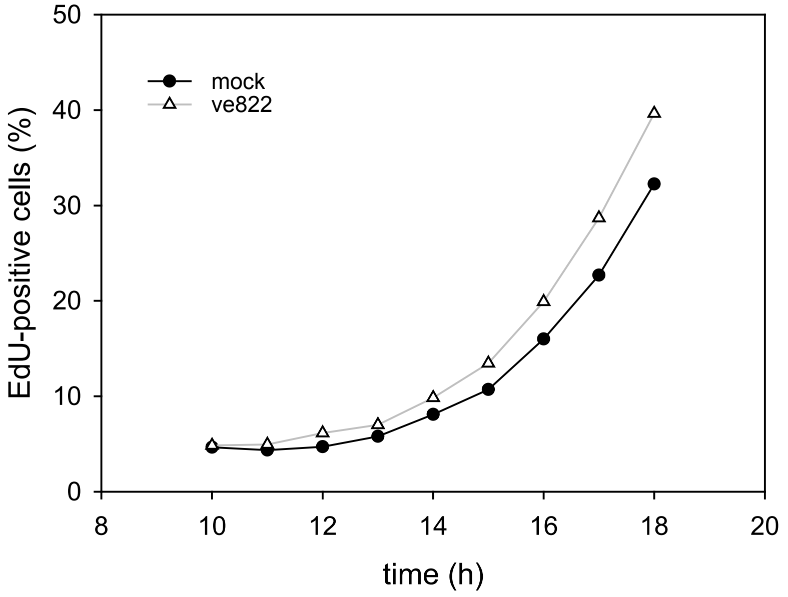

**Supplementary Figure S7**

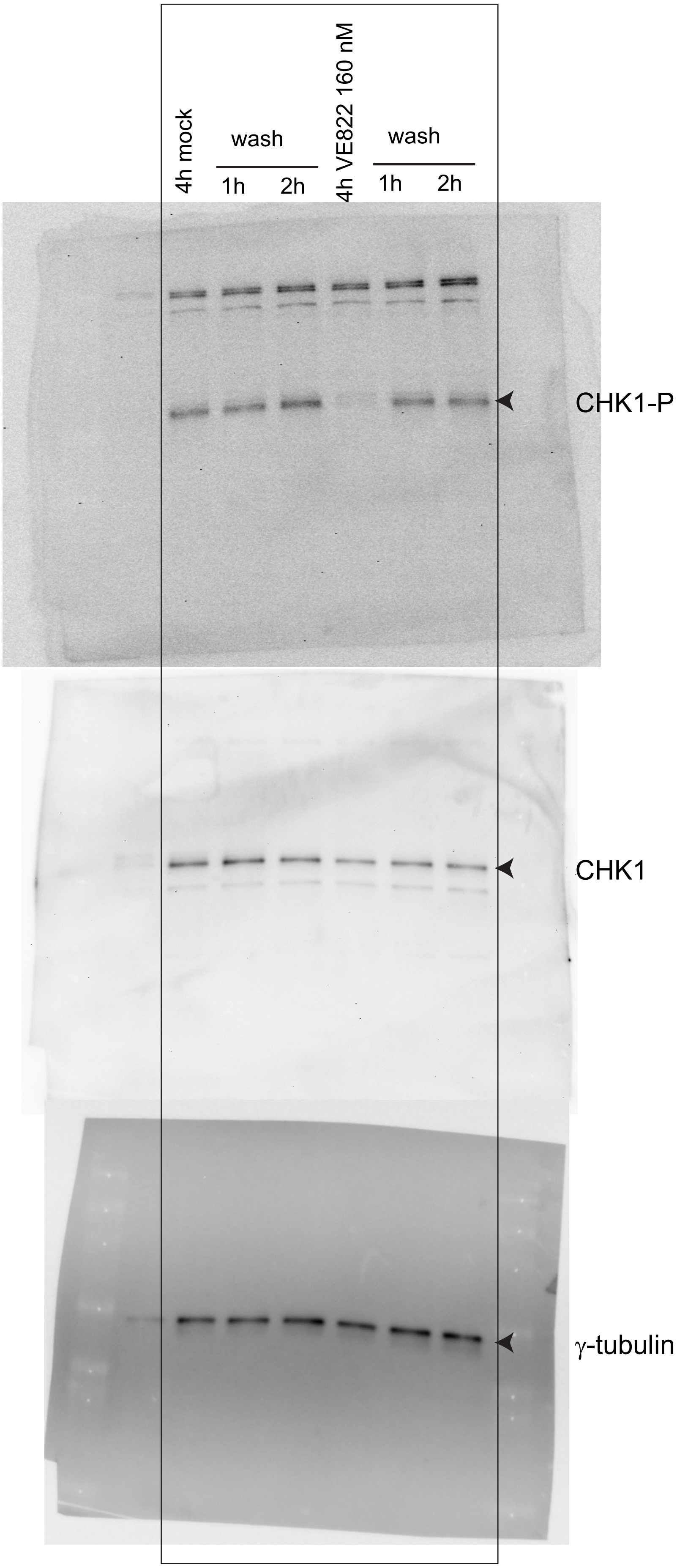

## Supplementary figure legends

*Supplementary Figure S1*

Full-length blots of the immunoblots shown on Fig 1C.

*Supplementary Figure S2*

Full-length blots of the immunoblots shown on Fig 2A.

*Supplementary Figure S3*

Full-length blots of the immunoblots shown on Fig 2D.

*Supplementary Figure S4*

Quantification of EdU-positive U2OS cells at the indicated time points after shake off of mitotic cells. 160nM ve822 ( ) or mock (DMSO) were added 1 h after release, for 4 h and the number of EdU-positive cells were counted by microscopy. A representative experiment is shown.

*Supplementary Figure S5*

Quantification of CDC45 and MCM signal intensity in G1 cells by flow cytometry.
**A.** CDC45 levels in extracted and not-extracted cells.
**B**. Gating for CDC45 positive cells
**C.** Gating for MCM3-positive cells.
**D.** DNA histograms.

*Supplementary Figure S6*

Analysis of EdU incorporation in BJ cells synchronized in G0, released into the cell cycle and treated with ATR inhibitor. Each time point was performed in separate barcoding sets including a sample from exponentially growing cells not given EdU (-EdU control). Average of 2 experiments is shown.

*Supplementary Figure S7*

Full-length blots of the immunoblots shown on Fig 6A.

## Table S1. Fission yeast strains used in this study

| Strain | Genotype | Source |
|---|---|---|
| 489 | cdc10-M17 | Paul Nurse |
| 1353 | cdc10-M17 rad3::ura4+ ura4-D18 | Lab collection |
| 1226 | cdc10-M17 mcm2:GFP:kanR ura4-D18 ? | Lab collection |
| 1407 | cdc10-M17 rad3::ura4+ mcm2:GFP:kanR ura4-D18 | Lab collection |
| 1253 | cdc10-M17 cdt1:cdt1-TAP:kanR ade6-M210 ura4-D18 leu1-32 | Lab collection |
| 1902 | cdc10-M17 cdt1:cdt1-TAP:kanR rad3::ura4+ | This work |
